# Listening in Naturalistic Scenes: What Can Functional Near-Infrared Spectroscopy and Intersubject Correlation Analysis Tell Us About the Underlying Brain Activity?

**DOI:** 10.1177/2331216518804116

**Published:** 2018-10-22

**Authors:** Stephen C. Rowland, Douglas E. H. Hartley, Ian M. Wiggins

**Affiliations:** 1 National Institute for Health Research Nottingham Biomedical Research Centre, UK; 2 Hearing Sciences, Division of Clinical Neuroscience, School of Medicine, University of Nottingham, UK; 3 Medical Research Council Institute of Hearing Research, School of Medicine, University of Nottingham, UK; 4 Nottingham University Hospitals NHS Trust, Queens Medical Centre, UK

**Keywords:** fNIRS, listening effort, virtual acoustics, intersubject correlation

## Abstract

Listening to speech in the noisy conditions of everyday life can be effortful, reflecting the increased cognitive workload involved in extracting meaning from a degraded acoustic signal. Studying the underlying neural processes has the potential to provide mechanistic insight into why listening is effortful under certain conditions. In a move toward studying listening effort under ecologically relevant conditions, we used the silent and flexible neuroimaging technique functional near-infrared spectroscopy (fNIRS) to examine brain activity during attentive listening to speech in naturalistic scenes. Thirty normally hearing participants listened to a series of narratives continuously varying in acoustic difficulty while undergoing fNIRS imaging. Participants then listened to another set of closely matched narratives and rated perceived effort and intelligibility for each scene. As expected, self-reported effort generally increased with worsening signal-to-noise ratio. After controlling for better-ear signal-to-noise ratio, perceived effort was greater in scenes that contained competing speech than in those that did not, potentially reflecting an additional cognitive cost of overcoming informational masking. We analyzed the fNIRS data using intersubject correlation, a data-driven approach suitable for analyzing data collected under naturalistic conditions. Significant intersubject correlation was seen in the bilateral auditory cortices and in a range of channels across the prefrontal cortex. The involvement of prefrontal regions is consistent with the notion that higher order cognitive processes are engaged during attentive listening to speech in complex real-world conditions. However, further research is needed to elucidate the relationship between perceived listening effort and activity in these extended cortical networks.

## Introduction

People with hearing loss report experiencing greater listening effort than their normally hearing peers (Alhanbali, Dawes, Lloyd, & Munro, 2017). Although listening effort may partly arise from hearing loss reducing the fidelity with which auditory signals are transduced within the ear, adverse acoustic conditions routinely encountered in daily life exacerbate the problem (Mattys, Davis, Bradlow, & Scott, 2012). Indeed, listening can be effortful even for people with normal hearing when a target signal is degraded through the presence of background noise (Zekveld, Kramer, & Festen, 2010), competing speech (Koelewijn, Zekveld, Festen, & Kramer, 2012), or room reverberation (Holube, Haeder, Imbery, & Weber, 2016). Listening effort has been the focus of increased research interest over recent years (McGarrigle et al., 2014; Pichora-Fuller et al., 2016), leading to advances in our understanding of the acoustic, linguistic, contextual, and individual factors that influence listening effort as well as the underlying neurocognitive processes. However, much is still to be learnt, including how listening effort is deployed in the complex and dynamic environments that characterize real-world listening. In this study, we aimed to combine the optical brain-imaging technique functional near-infrared spectroscopy (fNIRS) with real-world binaural stimuli to investigate perceived listening effort and associated brain activity in naturalistic acoustic scenes.

Recent articles have sought to provide clarity regarding the definition of listening effort. McGarrigle et al. (2014, p. 434) defined it as “the mental exertion required to attend to, and understand, an auditory message.” Subsequently, Pichora-Fuller et al. (2016, p. 5S) provided a more technical definition of effort as “the deliberate allocation of mental resources to overcome obstacles in goal pursuit when carrying out a task, with listening effort applying more specifically when tasks involve listening.” A variety of factors can make for adverse listening conditions (Mattys et al., 2012), with consequent increases in the amount of mental resources needed to successfully comprehend speech. Whether an individual will expend the required effort to achieve this goal depends on their level of motivation, which is in turn influenced by an internal assessment of success importance (Pichora-Fuller et al., 2016). From a clinical perspective, there is concern that chronically elevated listening effort could lead to fatigue, with associated negative effects on quality of life (Hornsby, Naylor, & Bess, 2016). Furthermore, excess listening effort could potentially contribute to accelerated cognitive decline in older adults with hearing loss, either through long-term impacts on brain structure or function (Cardin, 2016) or mediated by social isolation (Pichora-Fuller, Mick, & Reed, 2015). Given these serious potential consequences, a diverse array of subjective, behavioral, and physiological measurement approaches has been advanced to further our understanding of listening effort (McGarrigle et al., 2014). At present, however, it is unclear how various putative measures of listening effort relate to one another, with speculation that different measures may tap into different aspects of listening effort, including demands on perceptual or linguistic processing, working memory, and conflict monitoring and executive control networks, general alertness or arousal, vigilance, and acute stress when task demands outstrip available resources (McMahon et al., 2016; Miles et al., 2017; Pichora-Fuller et al., 2016).

An important goal for research is to reveal more about the neurocognitive mechanisms that underpin listening effort (Pichora-Fuller et al., 2016). Previous neuroimaging studies have revealed a general trend toward increased recruitment of frontal brain regions as listening conditions become more challenging, typically interpreted as reflecting an increase in the amount of cognitive effort being exerted (Adank, 2012; Davis & Johnsrude, 2003, 2007; Eckert, Teubner-Rhodes, & Vaden, 2016; Golestani, Hervais-Adelman, Obleser, & Scott, 2013; Hervais-Adelman, Carlyon, Johnsrude, & Davis, 2012; Peelle, 2018; Vaden, Kuchinsky, Ahlstrom, Dubno, & Eckert, 2015; Vaden et al., 2013; Wijayasiri, Hartley, & Wiggins, 2017; Wild, Davis, & Johnsrude, 2012a; Wild et al., 2012b; Zekveld, Heslenfeld, Festen, & Schoonhoven, 2006; Zekveld, Heslenfeld, Johnsrude, Versfeld, & Kramer, 2014). The recruited frontal regions subserve a variety of functional purposes. Recruitment of areas traditionally associated with speech production, for example, left inferior frontal gyrus (Broca’s area) and premotor cortex, could reflect the activation of motor speech representations or increased reliance on verbal working memory to support the comprehension of degraded speech (Hervais-Adelman et al., 2012; Peelle & Wingfield, 2016a; Wijayasiri et al., 2017; Wild et al., 2012b; Zekveld et al., 2006). Elevated activation within the cingulo-opercular system is thought to reflect self-monitoring of performance and the optimization of behavior taking into account the anticipated reward of completing an effortful task (Eckert et al., 2016; Vaden et al., 2013, 2015). Recruitment of dorsolateral prefrontal cortex, part of the domain-general frontoparietal attention network (Dosenbach, Fair, Cohen, Schlaggar, & Petersen, 2008), could reflect upregulation of top-down cognitive control mechanisms to help focus attention on a target sound in the presence of competing information (Eckert et al., 2016; Evans, McGettigan, Agnew, Rosen, & Scott, 2016; Sharp, Scott, Mehta, & Wise, 2006). A challenge for ongoing research is to better understand the specific listening conditions under which each of these supporting neural systems is recruited (Peelle & Wingfield, 2016a).

With a few notable exceptions (Damian, Corona-Strauss, Hannemann, & Strauss, 2015; Wu et al., 2014), most studies of listening effort have used laboratory-based tasks that represent the challenges of real-world listening in a highly simplified form. Thus, relatively little is known about how listening effort is deployed in the complex and dynamic environments of everyday life. In recent years, there has been a growing trend in neuroscience toward studying how the brain responds to complex, naturalistic stimuli (Hasson, Malach, & Heeger, 2010; Maguire, 2012). This necessitates the application of new analysis approaches that do not depend on a precise, parameterized description of the stimulus, nor an explicit model of how the brain will respond to that stimulus. One such approach is intersubject correlation (ISC) analysis (Hasson, Nir, Levy, Fuhrmann, & Malach, 2004). In the ISC approach, brain activity is measured in a group of participants who are exposed to an identical stimulus, and the analysis proceeds by identifying regions of the brain that respond similarly across participants. The approach has been used extensively in functional magnetic resonance imaging (fMRI) studies to investigate how the brain responds to complex stimulation, for example, during free viewing of movies or listening to audio books or music (Boldt et al., 2013; Hasson, Furman, Clark, Dudai, & Davachi, 2008; Hasson et al., 2004; Jääskeläinen et al., 2008; Jääskeläinen et al., 2016; Kauppi, Jääskeläinen, Sams, & Tohka, 2010; Nummenmaa et al., 2012; Trost, Fruhholz, Cochrane, Cojan, & Vuilleumier, 2015). In the auditory domain, Wilson, Molnar-Szakacs, and Iacoboni (2008) have shown that ISC has greater power to detect activation in higher level frontal brain regions during narrative comprehension than does conventional subtraction analysis (i.e., contrasting periods of listening vs. rest). Because listening under effortful conditions is also thought to recruit higher level frontal regions, ISC-based analyses could provide a powerful means to investigate this effort-related brain activity. Furthermore, recent studies have implemented a dynamic form of ISC analysis, which allows the momentary strength of intersubject brain synchronization to be related to time-varying properties of the stimulus, or participants’ subjective reaction to it (Nummenmaa et al., 2012; Trost et al., 2015). Adopting a similar approach in the present context could help to shed light on how specific cortical resources are recruited as the demands of a naturalistic listening situation unfold over time.

In this study, we investigated effortful listening to continuous speech narratives in naturalistic scenes. Virtual acoustics techniques were used in conjunction with binaural recordings made in a variety of real-world locations to recreate complex listening environments featuring ecologically relevant sources of background noise, competing speech, and room reverberation. We studied normally hearing participants’ subjective experiences of listening effort in these realistic scenes, alongside their brain activity. Brain activity was measured using the optical technique fNIRS. A silent and flexible alternative to fMRI, fNIRS holds promise for investigating the brain activity underlying effortful listening in naturalistic conditions (Peelle, 2017; Wijayasiri et al., 2017). Like fMRI, fNIRS measures the hemodynamic response to brain activation (Scholkmann et al., 2014). However, fNIRS achieves this by optical means; specifically, by noninvasively illuminating the brain with infrared light via optodes placed in contact with the scalp. Because the penetration depth of infrared light through biological tissue is limited, fNIRS measurements are strongly biased toward parts of the brain that are close to the scalp (Strangman, Li, & Zhang, 2013). Thus, fNIRS is unlikely to be suitable for probing all regions implicated in effortful listening, with much of the cingulo-opercular system, for example, lying too deeply to be accessible. However, other relevant regions, such as the inferior frontal gyrus and dorsolateral prefrontal cortex, are in principle amenable to imaging by optical means.

Our primary aim was to establish the viability of using ISC analysis on fNIRS data to identify cortical regions involved in comprehending narrative speech in naturalistic scenes. Based on prior fMRI findings (Honey, Thompson, Lerner, & Hasson, 2012; Lerner, Honey, Silbert, & Hasson, 2011; Schmälzle, Häcker, Honey, & Hasson, 2015; Wilson et al., 2008), we anticipated that ISC analysis would reveal the involvement of an extended network of frontotemporal brain regions. We additionally aimed to examine the interrelationships among self-reported listening effort, brain activity, and simple acoustic descriptors of the auditory scenes. Specifically, we considered better-ear signal-to-noise ratio (SNR) as a proxy for scene-by-scene acoustic difficulty, as well as the envelopes of the target speech and background sound to test whether brain activity tracked moment-to-moment fluctuations in the acoustic energy of the constituent sound sources.

## Methods and Materials

### Overview

Each participant attended a single session, during which testing took place in a double-walled sound booth. Participants first listened to a set of speech narratives while having their frontotemporal brain activity measured using fNIRS. Participants were instructed to maintain attention on a target talker while the auditory scene smoothly transitioned in the background, varying the nature and degree of the acoustic challenge. To encourage sustained attention to the target speech, participants were asked a series of multiple-choice questions relating to the content of each narrative immediately after hearing it. Participants subsequently listened to another set of closely matched narratives and rated subjective effort and intelligibility for each scene in turn. Before each stage of the experiment, participants completed a short practice run to gain familiarity with the relevant task.

### Participants

Thirty university students aged 18 to 23 years (17 women) took part in the study. All participants had (self-reported) normal hearing, normal or corrected-to-normal vision, and no known cognitive or motor impairments. They were all native English speakers. Of the 30 participants, 22 were right handed, four were left handed, and four showed no strong preference for either hand, as assessed using the Edinburgh Handedness Inventory (Oldfield, 1971). The study was approved by the University of Nottingham Faculty of Medicine and Health Sciences Research Ethics Committee, and written informed consent was obtained from all participants.

### Stimuli

Stimulus materials were adapted from the RealSpeech™ content library (used with permission of Dr. Ian Wiggins and Dr. Mark Fletcher). This library provides a collection of (a) target-speech narratives recorded under near-anechoic conditions (dry speech signal), (b) real-world binaural recordings of background sounds made in a variety of everyday listening environments, and (c) the means to flexibly combine these such that a chosen talker can be simulated speaking in a particular environment (with the acoustics of that environment accurately recreated through convolution of the dry speech signal with an appropriate binaural room impulse response). The real-world recordings were made using a proprietary binaural microphone with realistic artificial pinnae. Note that because the target-speech narratives were separately recorded in a quiet environment, there was no recreation of the Lombard effect (the involuntary increase in vocal amplitude that might have been expected to occur had the speech truly been produced in a noisy environment).

In this study, as target speech, we used three narratives for brain imaging and a further three narratives for the subsequent subjective-ratings task. These narratives covered a variety of general-interest topics (e.g., the use of pit ponies in the mining industry from the mid-18th until the mid-20th century; the changing climate on Mars; a biography of English musician John Lennon, co-founder of the Beatles) and were each of approximately 4 to 5 min duration.

As background scenes, we used a total of 17 different environments. These included a busy café, an open-plan office, a swimming pool, various residential rooms and outdoor spaces, and a traveling car. The use of binaural recordings allowed us to approximate real-world spatial sound fields by presenting the stimuli to participants through insert earphones (Hammershøi and Møller, 2005).

To present participants with a continually varying acoustic challenge, we generated stimuli in which the background auditory scene smoothly transitioned every 8 to 27 s (median 13.8 s), timed to coincide with natural pauses in the narratives. This was achieved by crossfading between scenes (3-s crossfade duration). The SNR within each scene was manually set during study design with the aim of achieving a suitably wide range of listening difficulties overall (SNR range across scenes: −16 dB to +25 dB). Subsequently, the scene-specific SNRs were fixed across participants such that everyone was presented with the same stimuli. Each narrative comprised between 18 and 23 scenes.

The stimuli were presented binaurally through Etymotic ER-2 insert earphones (Etymotic Research, Inc., Elk Grove Village, Illinois). Prior to the main experiment, participants were played a representative sample of target speech (in quiet), followed by one of the loudest scenes, and given the opportunity to adjust the overall volume to a comfortable level. Participants could not make any adjustment to the SNR using this process. Because the ER-2 earphones are designed to achieve a flat frequency response at the human eardrum, yet the source recordings were made using the equivalent of a blocked-entrance microphone position, it was necessary to filter the stimuli to reinstate the main ear-canal resonance. We applied the required broad mid-frequency boost based on group-average data provided by Hammershoi and Moller (1996). We corrected only up to 8 kHz, reasoning that individual variability at higher frequencies would render any attempt at correction ineffective. It must be noted that the use of generic, rather than individualized, binaural stimuli, together with the absence of individualized earphone equalization, will have limited the fidelity of the virtual acoustic reproductions (Begault, Wenzel, & Anderson, 2001; Wightman & Kistler, 2005; Xu, Li, & Salvendy, 2007). We cannot be sure to what extent each participant experienced a true-to-life spatial auditory percept. Nevertheless, participants were exposed to naturalistic speech-in-noise stimuli capable of evoking a general sense of spaciousness and containing interaural cues of physiologically plausible magnitude.

**Figure 1.**
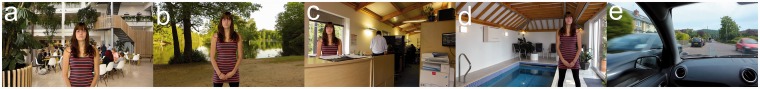
Examples of real-world listening environments recreated in this study. A range of indoor (e.g., scenes (a), (c), and (d)) and outdoor (e.g., scene (b)) scenes were used. A mixture of competing speech (e.g., scenes (a) and (c)), ambient background sound (e.g., scenes (b) and (e)), and adverse room acoustics (e.g., scenes (a) and (d)) presented a wide variety of acoustic challenges.

During listening, participants were presented with a still image on a computer display that represented the auditory environment currently being simulated, including the location of the target talker within the scene (Figure 1). The visual images were smoothly cross-faded in time with the auditory scene transitions. This visual information was provided to help participants remain orientated within the simulated sound fields, considered important in this study given the complex nature of the real-world listening environments being recreated and the fact that the background scene changed regularly while the listening task was ongoing. Because neither the talker nor the background was animated, the accompanying visual stimulation did not provide speech-reading cues. Nonetheless, the visual context provided by these images may still have benefitted speech understanding, by, for example, helping the listener to understand the nature of the interfering sounds or cueing the spatial location of the target talker. Indeed, it has been shown that prior knowledge of a target talker’s location can significantly improve performance in complex listening scenarios (Kidd, Arbogast, Mason, & Gallun, 2005; Kitterick, Bailey, & Summerfield, 2010).

### Brain-Imaging and Content-Recognition Task

Three narratives were presented sequentially during brain imaging, each read by a different talker (two men and one woman). The order of presentation of the three narratives was randomized for each participant, though within each narrative all participants received identical stimulation (order of scenes and SNR within each scene fixed across participants). Each narrative was preceded by a 10-s title screen and immediately followed by a series of multiple-choice “fill in the blank” style questions designed to assess recognition of the content just heard. The questions comprised short extracts from the transcript of the relevant narrative with a word missing, together with four options for what the missing word might have been. The three foils were in each case chosen to be semantically plausible; however, the choice of foils was not guided by any formal evaluation of phonological or semantic similarity or word frequency. There was typically one question per scene and questions were presented in the same order as the associated content had appeared in the auditory narrative. The questions were presented on the computer display and participants responded by using a mouse to select the correct word. To ensure that the experimental session moved along at a consistent pace, participants were given a fixed time window of 30 s to answer as many questions as possible. To promote task engagement, participants were provided with feedback on the number of questions they answered correctly (6 s); there was then a 10-s pause before the title screen for the next narrative appeared. Brain activity was continuously measured using fNIRS throughout.

### Subjective-Ratings Task

The subjective-ratings task followed a similar format to the brain-imaging task, except with each scene now presented in isolation rather than continuously flowing one into the next. This gave time for participants to provide their subjective ratings of effort and intelligibility immediately after listening to each scene. Effort was probed with the question “How effortful was it to follow what the talker was saying?” and responses were given on a 10-cm visual analog scale with end points labeled “Not at all effortful” and “Extremely effortful.” Participants could respond anywhere along the scale, and there were no intermediate marks or labels. Similarly, intelligibility was probed with the question “How much of what the talker said could you understand?”, and the visual analog scale end points were labeled “Not a word” and “Every single word.” Effort and intelligibility were jointly probed on the same screen to encourage participants to recognize these as distinct perceptual dimensions. In addition to the effort and intelligibility scales, the response screen included a box which participants were instructed to tick if, and only if, they had found listening in a given scene so difficult that they had felt compelled to give up. Participants still had to give effort and intelligibility ratings, even if ticking this box. The task was automated and participants responded using a mouse as the input device.

Different narratives were used for the subjective ratings, compared with those used for brain imaging, to avoid familiarity effects. However, for each narrative used during brain imaging, a corresponding narrative was prepared for the subjective-ratings task that was as closely matched as possible. Paired narratives were matched in the following ways: (a) the identity of the talker was the same; (b) the duration of the target speech within each scene was matched as closely as natural breaks within the speech would allow; and (3) within each scene, the A-weighted root mean square level of the target speech was equalized between the corresponding brain-imaging and subjective-ratings narratives.

### fNIRS Data Acquisition

**Figure 2.**
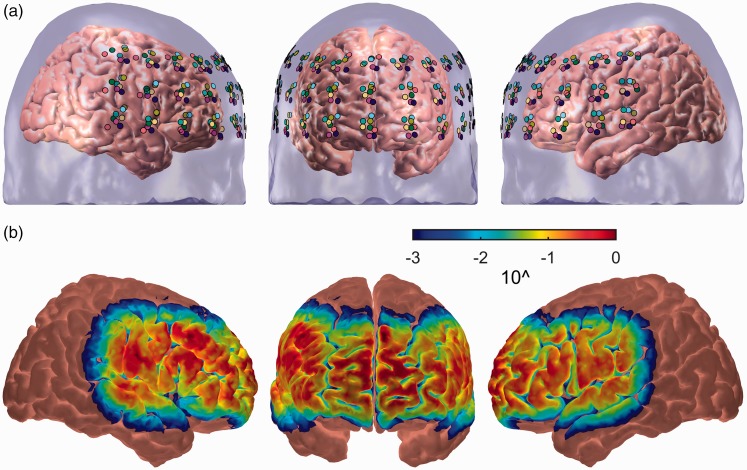
(a) Variation in optode positioning across eight volunteers (color-coded according to individual). These data were acquired using a three-dimensional digitizer to record optode positions, as well as anatomical surface landmarks, followed by registration to a standard atlas brain. (b) Aggregate cortical sensitivity profile across the entire probe-set calculated using the AtlasViewer tool (Aasted et al., 2015). The color scale depicts relative sensitivity on a logarithmic scale from 0.001 to 1.

The fNIRS data were acquired using a Hitachi ETG-4000 system (Hitachi Medical Co., Japan). The ETG-4000 is a continuous-wave system that measures simultaneously at wavelengths of 695 and 830 nm at a sampling rate of 10 Hz. A 3 × 11 optode array comprising 17 emitters and 16 detectors was used providing coverage of prefrontal and (superior) temporal cortex (Figure 2). There were 52 measurement channels in total.

## Analyses

### fNIRS Data Preprocessing

Preprocessing of the fNIRS data was performed in MATLAB (MathWorks, Natick, MA) and proceeded along similar lines to previous fNIRS studies conducted in our laboratory (Anderson, Wiggins, Kitterick, Hartley, 2017; Wiggins, Anderson, Kitterick, & Hartley, 2016; Wiggins & Hartley, 2015; Wijayasiri et al., 2017). First, the raw intensity signals were converted to changes in optical density (Huppert et al., 2009). Then, motion-artefact correction was applied using a wavelet filtering approach (Molavi & Dumont, 2012) with an exclusion threshold for wavelet coefficients of 0.719 times the interquartile range below the first quartile or above the third quartile. The data were then bandpass filtered between 0.01 and 0.5 Hz to remove low-frequency drift and cardiac oscillations. Finally, a hemodynamic signal separation method was applied to help isolate the functional response to brain activation from the systemic physiological signals that can otherwise confound fNIRS measurements (Yamada, Umeyama, & Matsuda, 2012). Subsequent statistical analyses were performed based on the estimated functional component of the hemodynamic signal.

Prior to statistical analysis, we excluded channels suffering from poor contact between optode and scalp. We identified such channels using the scalp-coupling index described by Pollonini et al. (2014). We excluded channels with scalp-coupling index < 0.12; this threshold was chosen to exclude only the worst 5% of channels across the entire dataset.

For each participant, data from the three narratives were placed into a uniform order, demeaned, and concatenated. The first 6 s of each narrative, corresponding to the fade-in period, were trimmed prior to concatenation. Subsequent analyses were performed on the concatenated data.

### fNIRS Data Analysis

#### Long-term ISC analysis

Our primary approach to analyzing the fNIRS data was to test for overall similarity of brain activation across individuals by computing the ISC between every pair of participants (Hasson et al., 2004). Following Wilson et al. (2008), prior to calculating ISC, we regressed out the global mean signal (i.e., the average time course across all fNIRS measurement channels) from each channel: The aim of doing so was to minimize the influence of any common, spatially nonselective brain activity across individuals, as might have resulted from changes in arousal, heart rate, and so forth. Where a given channel had been excluded for a certain participant, that participant was left out from the calculation of ISC for that channel (i.e., the effective sample size was reduced by one). Because the correlation coefficient *r* is not normally distributed, the Fisher *z* transformation was applied: *z* = 0.5 × ln((1 + *r*)/(1 − *r*)). To test whether mean ISC values were significantly greater than zero, we performed one-tailed *t* tests on a channel-wise basis (Wilson et al., 2008).

#### Time-varying ISC analysis

We were also interested to explore whether the momentary strength of ISC varied systematically according to short-term listening conditions. This could have occurred if, for example, activity in a certain brain region became more strongly synchronized across participants during periods when listening was particularly effortful. Note that such changes in synchronicity across participants can in principle occur even in the absence of any overall increase or decrease in the level of activity in a particular brain region. To this end, following Nummenmaa et al. (2012), we performed a sliding-window dynamic ISC analysis over the full duration of the concatenated data. The window size was 16 s and the step size was 2 s. The ISC was calculated on the windowed data using the same procedure as described earlier, resulting in a mean ISC value per window per fNIRS channel. We then used general linear model (GLM) analyses to test whether momentary ISC could be explained by relevant subjective or acoustic descriptors of the time-varying listening conditions, as described later.

#### GLM analyses

We used GLM analyses to test for relationships between various subjective or acoustic predictors and brain activity. We conducted two types of GLM analyses. In the first type, introduced earlier, we attempted to model time-varying ISC in a similar manner to Nummenmaa et al. (2012). In these models, predictor variables were time aligned with the fNIRS data by convolution with a simple Gaussian filter (5-s time-to-peak). The second type of GLM analyses was of a more conventional nature, in that they aimed to model the magnitude of brain activation directly (Bartels & Zeki, 2004; Evans et al., 2016); that is, these models tested for brain regions in which the level of activity systematically increased or decreased as listening became more or less effortful, for example. In these models, predictor variables were convolved with the canonical dual-gamma hemodynamic response function (HRF) provided in SPM8 [http://www.fil.ion.ucl.ac.uk/spm]. In both types of GLM, we applied the Cochrane-Orcutt procedure recursively to deal with serial correlation in the error term (Cochrane & Orcutt, 1949). Our stopping criterion was when the autocorrelation in the model residuals changed by less than 0.01 between one iteration and the next. In the case of models aiming to predict the magnitude of brain activation directly, we took the beta weights from the individual-participant models to a group-level random effects analysis by performing two-tailed *t* tests on a channel-wise basis.

As potential predictors of brain activity, our primary variables of interest were participants’ ratings of listening effort and intelligibility. That is, we looked to explain brain activity in terms of subjective experiences associated with effortful listening. It is important to note that *perceived* effort (the subjective experience of how taxing a task is or was) does not necessarily equate to *processing* effort (the mental resources deliberately allocated to overcome obstacles in goal pursuit when carrying out a task) (Lemke & Besser, 2016; Pichora-Fuller et al., 2016). Nonetheless, we anticipated that there would be systematic relationships between brain activity and subjective ratings of listening effort or intelligibility. Across multiple other studies that employed complex, naturalistic stimuli, brain activity has been found to be predicted by subjectively rated experiences, including humorousness (Franklin & Adams, 2011; Jääskeläinen et al., 2016) and emotional valence or arousal (Nummenmaa et al., 2012, 2014; Trost et al., 2015; Viinikainen et al., 2012). When modeling time-varying ISC, we used group-average ratings of listening effort or intelligibility, while when modeling the magnitude of brain activation directly, we used each individual’s subjective ratings to predict their own brain response. As an objective acoustic metric, we also considered the scene-by-scene SNR at whichever ear received the more favorable look at the target speech (better-ear SNR). Recall that the SNR was fixed across participants, and in calculating better-ear SNR, we used the equivalent A-weighted level of the speech and background sound averaged over the duration of each scene (see Supplementary Figure S1 for details of the distribution of better-ear SNR across scenes and narratives). Finally, we included the instantaneous amplitude envelopes of the target speech and background sound (separated from one another acoustically). This allowed us to test for brain responses that directly tracked the envelope of the acoustic signals, as opposed to higher level concepts such as the scene-by-scene SNR. In extracting the envelopes, the signals were passed through an A-weighting filter, and the overall level across the two ears was calculated assuming energetic summation. The envelopes were temporally smoothed through convolution with the canonical HRF.

#### Correction for multiple comparisons across channels

As this is, to the best of our knowledge, the first attempt to probe brain activation during effortful listening in naturalistic scenes, we adopted an exploratory approach, testing across the entire probe-set in a channel-wise manner, rather than in predefined regions of interest. To correct for the multiple comparisons issue associated with performing simultaneous testing in 52 measurement channels, we controlled the false discovery rate following the procedure described by Benjamini and Hochberg (1995).

### Subjective-Ratings Analysis

Following a similar approach to Bartels and Zeki (2004), we used correlational analyses to establish: (a) the degree of consistency between participants in their subjective ratings of listening effort and intelligibility and (b) the relationship between self-reported effort and intelligibility within individuals. To assess consistency between participants, we calculated the Pearson’s correlation coefficient between effort (or intelligibility) ratings from all possible pairs of participants, on a per-narrative basis. To assess the relationship between self-reported effort and intelligibility, we calculated the correlation between these percepts on a within-participant basis, before averaging across participants. In all cases, we applied the Fisher *z* transformation to the individual correlation coefficients before calculating means and standard deviations. Results were converted back to the original correlation scale for ease of interpretation.

To explore what drives self-reported effort and intelligibility, we plotted the respective mean ratings against better-ear SNR for each scene. We performed broken-line fits to better understand the nature of these relationships. We also assessed whether self-reported effort or intelligibility depended on the presence of competing speech. We manually categorized each scene according to whether the background (when listened to in isolation) featured potentially intelligible speech of an ongoing nature. Scenes containing only isolated vocalizations, or vocalizations that were clearly unintelligible (e.g., distant laughter), were classed as not containing competing speech. Across all narratives, 16 of 63 scenes (≈25%) were categorized as containing competing speech. We used multiple linear regression to test whether self-reported effort (or intelligibility) was significantly different in those scenes that contained competing speech compared with those that did not, after controlling for better-ear SNR. Better-ear SNR was controlled for statistically since it was not precisely matched between categories (up to 3 dB difference in mean better-ear SNR, depending on the narrative).

## Results

### Content-Recognition Task Run Alongside the Brain-Imaging Data Collection

The mean number of questions answered (across narratives and participants) was 5.3, with a mean success rate of approximately 50% (chance performance would have been at 25% correct). The relatively low success rate might stem from participants having had to retain details of each narrative over a duration of several minutes. Indeed, due to the limited number of questions that participants managed to answer within the time-limited period (corresponding to around 25%–30% of all available questions), they primarily responded to questions on content that had appeared toward the start of the narrative, several minutes before the questions were posed. Of course, the fact that listening was, by design, highly challenging during a proportion of each narrative will also have limited participants’ success rates. A limitation arising from the relatively small proportion of questions that participants were able to complete within the time limit is that, in practice, they were not probed on their retention of details relating to the mid and latter parts of each narrative. As this prevents the use of these data to provide an indication of the extent to which an individual sustained engagement throughout the full listening task, these results are not considered further.

### Subjective Ratings

Table 1 shows the results of the subjective-ratings analysis. Participants were generally consistent with one another in their ratings of effort (mean *r* across narratives = .81) and intelligibility (mean *r* = .82). Within participants, self-reported effort and intelligibility were strongly negatively correlated (mean *r* = −.83). In all cases, correlation values were similar across the three narratives, suggesting that these relationships generalize across different talkers and speech materials.

**Table 1.** Correlation Coefficients (Mean ± *SD*) Among Subjective Ratings of Listening Effort and Intelligibility Between Participants (Top Two Rows) and Between Percepts Within Participants (Bottom Row).

	Narrative 1	Narrative 2	Narrative 3
Correlation of effort across all pairs of participants	0.81 ± 0.24	0.82 ± 0.26	0.80 ± 0.09
Correlation of intelligibility across all pairs of participants	0.83 ± 0.09	0.85 ± 0.10	0.78 ± 0.09
Correlation between effort and intelligibility ratings within participants	−0.81 ± 0.12	−0.84 ± 0.09	−0.85 ± 0.08

Participants rarely reported having felt compelled to give up listening because the target speech was too hard to follow. Depending on the narrative, no participants reported giving up in between 68% and 82% of all scenes. The greatest proportion of participants who reported giving up on any single scene was 7 of 30 (23%) for Scene 7 of Narrative 1. In conclusion, it seems that the stimuli presented participants with a suitable range of listening difficulties overall, except for possibly a small number of scenes that may have been too challenging for a minority of individuals.

**Figure 3.**
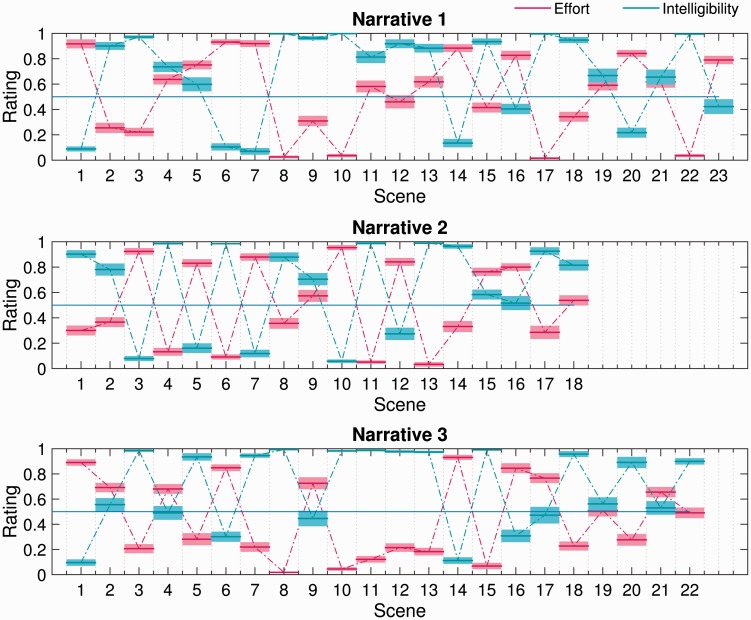
Scene-by-scene subjective ratings of effort and intelligibility for each narrative used in the subjective-ratings task. The mean rating across participants (bold horizontal lines) ±1 standard error of the mean (surrounding shaded regions) is shown in each case.

**Figure 4.**
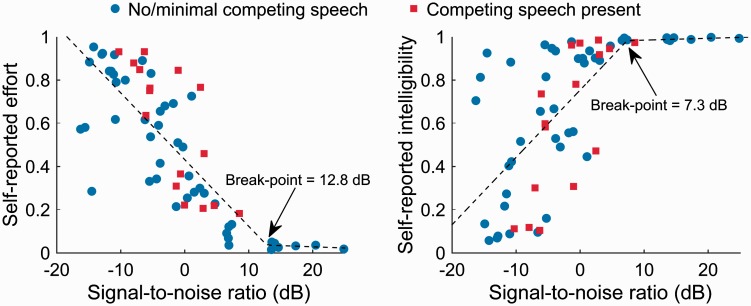
Self-reported effort (left) and intelligibility (right) as a function of better-ear signal-to-noise ratio. Marker style and color are used to differentiate scenes that contained competing speech (red squares) from those that did not (blue circles). Also, broken-stick fits to the data with the break points indicated are shown.

Figure 3 plots mean scene-by-scene effort and intelligibility ratings for each narrative used in the subjective-ratings task. Consistent with the correlational analyses, it is clear that self-reported effort generally increased as intelligibility decreased and vice versa. However, it is interesting to note that there are several examples of scenes (e.g., Narrative 1 Scenes 9 and 18; Narrative 2 Scene 14; Narrative 3 Scene 5) in which listening was reported to be moderately effortful, despite perceived intelligibility being near perfect. This observation is further supported by Figure 4, which plots self-reported effort and intelligibility against better-ear SNR. The broken-stick fits make clear that effort generally decreased, and intelligibility generally increased, as the SNR improved. However, above a certain SNR, listening became effortless and the target speech became fully intelligible. Interestingly, the break points in the fits suggest that perceived intelligibility became perfect some 5 dB or so before listening became subjectively effortless.

Multiple linear regression analysis showed that, after controlling for better-ear SNR, self-reported effort was significantly higher in scenes that contained competing speech than in those that did not, *t*(60) = 2.02, *p* = .048. There was no corresponding effect of competing speech on perceived intelligibility, *t*(60) = − 0.88, *p* = .381.

### fNIRS Results

**Figure 5.**
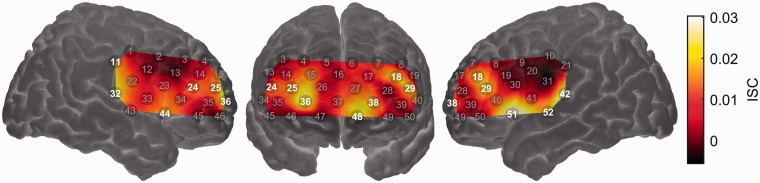
Intersubject correlation (ISC) map across the entire probe-set. Channels showing statistically significant ISC (*q* < 0.05, false discovery rate corrected) are highlighted. Note that the map is interpolated from single-channel results and the overlay on the brain is for illustrative purposes only. Brighter colors (yellow or white) indicate higher ISC, that is, more strongly synchronized brain activity across individuals.

Figure 5 shows the mean long-term ISC map across the entire probe-set. As expected, significant ISC (*q* < 0.05, false discovery rate corrected) was observed in channels overlying the bilateral auditory cortices (Channels 32, 42, and 52). However, significant ISC was not circumscribed to superior temporal cortex, with significant ISC also seen in channels overlying the right postcentral gyrus (Channel 11), bilateral inferior frontal cortex (Channels 44 and 51), bilateral dorsolateral prefrontal cortex (Channels 18, 24, and 29), and bilateral frontopolar cortex (Channels 25, 36, 38, and 48). Spatially extensive ISC during listening to complex auditory narratives is consistent with previous fMRI studies (Honey et al., 2012; Lerner et al., 2011; Schmälzle et al., 2015; Wilson et al., 2008).

In the GLM analyses aiming to model time-varying aspects of brain activity based on various subjective or acoustic predictors, we observed few significant results after controlling for multiple comparisons across measurement channels. Whether modeling time-varying ISC or the magnitude of brain activation directly, we did not find any statistically significant relationships between brain activity and (a) self-reported effort, (b) self-reported intelligibility, or (c) scene-by-scene better-ear SNR. Results for these analyses are therefore not plotted.

**Figure 6.**
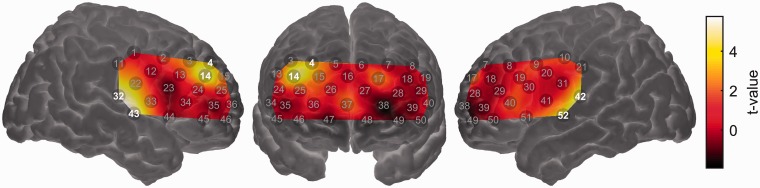
Group-level random effects analysis testing for a linear relationship between the (smoothed) time-varying envelope of the target speech signal and the strength of brain activation. Channels showing a statistically significant relationship (*q* < 0.05, false discovery rate corrected) are highlighted. Note that the map is interpolated from single-channel results and the overlay on the brain is for illustrative purposes only.

The one case in which we did observe significant relationships between an acoustical predictor and brain activity was when modeling the magnitude of brain activation using the smoothed amplitude envelopes of the target speech and background sound as predictors. These two predictors were entered into a GLM simultaneously such that it was possible to assess the effect of the target-speech envelope on brain activation while controlling for the effect of the background sound envelope and vice versa. Activation in channels overlying the bilateral auditory cortices (Channels 32, 42, 43, and 52) was significantly related to the target-speech envelope, suggesting that these regions responded principally to the presence of the target speech (Figure 6). The same was true for a region of right dorsolateral prefrontal cortex (Channels 4 and 14). No channels showed a response that was significantly driven by the envelope of the background sound (results not shown).

## Discussion

We combined the optical neuroimaging modality fNIRS with a model-free, data-driven analysis technique (intersubject correlation) to investigate brain activity associated with effortful listening to speech in naturalistic scenes. In the same individuals, we also investigated self-reported listening effort and intelligibility under similar conditions. Consistent with expectation based on prior imaging studies, the results indicate that higher order frontal brain regions are engaged during effortful listening in complex, real-world conditions. However, the degree to which specific brain regions are engaged in different listening environments, and as listening becomes more or less effortful, remains unclear. Our results additionally provide insight into the factors likely to influence perceived listening effort under ecologically relevant conditions.

### Attentive Listening to Speech in Naturalistic Scenes Elicits Common Brain Activity Across Individuals

The long-term ISC analysis identified several cortical regions that responded similarly across individuals during attentive listening to speech in naturalistic scenes. Unsurprisingly, these regions included bilateral superior temporal cortex, well known to play a fundamental role in speech processing (Hickok & Poeppel, 2007). Consistent with previous fMRI studies that employed ISC during narrative comprehension (Honey et al., 2012; Lerner et al., 2011; Schmälzle et al., 2015; Wilson et al., 2008), we additionally observed significant ISC across a swathe of prefrontal cortex. Significant ISC was observed not only in the core language network (e.g., superior temporal gyrus and inferior frontal gyrus) but also in extralinguistic regions (e.g., dorsolateral prefrontal cortex and frontopolar cortex). Common brain activity in these extralinguistic regions may reflect the processing of narrative meaning or social content over extended timeframes (Honey et al., 2012; Lerner et al., 2011), or, potentially, the time-varying demand placed upon domain-general cognitive control networks as the degree of acoustic challenge varied (Blank & Fedorenko, 2017; Regev, Honey, Simony, & Hasson, 2013).

These results confirm the viability of using ISC analysis on fNIRS data, a promising approach which has only just begun to be explored in the literature (Liu et al., 2017). This is important because ISC is a powerful technique for analyzing brain activity collected under complex, naturalistic conditions, while fNIRS is itself uniquely well suited among neuroimaging modalities for use in unconstrained, naturalistic settings (Balardin et al., 2017; Quaresima & Ferrari, 2016). Nonetheless, the results of ISC analyses can be difficult to interpret in isolation. Significant ISC in a given brain region does not necessarily imply an active role of that region in processing the experimental stimuli: Indeed, significant ISC can occur in regions that are deactivated during stimulus presentation compared with rest (Wilson et al., 2008). Significant ISC merely implies that a given brain region responds similarly across individuals to some fluctuating element of the stimulus or task, without offering insight into the functional role of that response. We therefore conducted a series of secondary analyses aimed at understanding how the brain responded to changes in listening difficulty across scenes.

### A Neural Marker for Real-World Listening Effort Remains Elusive

Using GLM analyses, we attempted to identify brain regions that responded systematically to variations in self-reported listening effort across scenes. We looked both for regions that showed an overall increase or decrease in activation under more effortful conditions (conventional analyses) and increased or decreased synchronization across individuals under more effortful conditions (dynamic ISC analyses). Based on prior research, candidate regions that might have been expected to show sensitivity to listening effort include: (a) the left inferior frontal gyrus, which has been shown to respond more strongly to degraded-yet-intelligible speech than to either clear speech or unintelligible noise (Davis & Johnsrude, 2003; Hervais-Adelman et al., 2012; Wijayasiri et al., 2017; Wild et al., 2012b; Zekveld et al., 2014); (b) prefrontal regions underlying the forehead, which have been shown using fNIRS to be sensitive to mental workload (Ayaz et al., 2012; Herff et al., 2013); and (c) fNIRS-accessible components of the domain-general “multiple demand” system (including dorsolateral prefrontal cortex) in which activity has been shown to scale with cognitive effort across a range of linguistic and nonlinguistic tasks (Blank & Fedorenko, 2017; Duncan, 2010). However, we did not find statistically significant relationships between self-reported effort and brain activity in any of these regions.

It is unclear why we did not observe significant relationships between brain activity and self-reported listening effort. One possibility is that this reflects the complexity of the relationship between “processing effort” (as quantified by physiological indices of listening effort, including brain activity) and “perceived effort” (as quantified by self-report) (Alhanbali et al., 2017). Indeed, ours is by no means the first study to find difficulty in establishing a relationship between self-reported listening effort and some objective measure of effort (McGarrigle et al., 2014). However, it is noteworthy that we were also unable to identify significant relationships between brain activity and an objective acoustic metric of listening difficulty (better-ear SNR). Self-reported listening effort, in contrast, was strongly (negatively) correlated with better-ear SNR.

A second possibility is that it is overly ambitious to seek a neural marker of listening effort that generalizes across the wide range of complex, naturalistic scenes included in this study. Different scenes presented different forms of acoustic challenge (adverse room acoustics, background noise, and competing speech), and it may be that different types of listening challenge recruit different neurocognitive processes to support successful comprehension (Peelle, 2018; Peelle & Wingfield, 2016b). Equally, it must be acknowledged that the scenes and narratives also varied in other, uncontrolled dimensions (e.g., level, spectral content, complexity of spectro-temporal modulations, spatial locations of target and background sound sources, and semantic content of the target speech and context of the background): Influences of these uncontrolled dimensions on brain responses may have obscured any systematic relationship with self-reported effort. Nonetheless, it is worth noting the remarkable success that others have had in decoding sensory experiences from neuroimaging data collected under similarly complex and uncontrolled conditions, albeit using more advanced machine-learning techniques (Floren, Naylor, Miikkulainen, & Ress, 2015; Valente, De Martino, Esposito, Goebel, & Formisano, 2011).

In a final analysis, we tested for brain regions in which activity was driven by the acoustic envelope of the target speech or background sound. We found that bilateral superior temporal cortex was significantly responsive to the envelope of the target speech. This result provides reassurance that the absence of significant effects in the analyses reported earlier is unlikely to have been due to a fundamental lack of statistical power. It is interesting that activity in fNIRS channels overlying superior temporal cortex tracked the envelope of the target speech but not the background sound. This apparent preference for speech, over acoustic stimulation in general, may reflect the depth profile of our fNIRS measurements. The primary auditory cortex (which would be more likely to track fluctuations in overall acoustic energy) lies hidden in the depth of the Sylvian fissure (Moerel, De Martino, & Formisano, 2014); our fNIRS measurements, biased toward more superficial parts of the brain (Strangman et al., 2013), likely had greater sensitivity to belt or parabelt regions, which respond preferentially to spectrotemporally complex sounds such as speech (Rauschecker & Scott, 2009). Interestingly, we found that a region of right dorsolateral prefrontal cortex was also significantly responsive to the envelope of the target speech. While further investigation is needed to clarify the precise nature of this response, it is possible that it reflects increased demand on cognitive control processes during periods of active listening (i.e., when the target speech was present; Sharp et al., 2006). It is noteworthy that a recent fMRI study of narrative speech perception in noise also observed right-lateralized prefrontal activation, specifically in response to sound onsets (Evans et al., 2016). These onset responses were interpreted as “phasic alerting” responses, preparing the listener for subsequent stimulation.

### Comprehension Succeeds Before Listening Becomes Effortless

Despite wide variability in acoustic conditions across the range of complex, naturalistic scenes included in this study, we found that both self-reported effort and intelligibility were strongly correlated with a simple acoustic metric of listening difficulty (better-ear SNR). Interestingly, our results suggest that while perceived intelligibility became perfect at a better-ear SNR of around +7 dB, listening did not become subjectively effortless until the better-ear SNR further improved to around +13 dB. Thus, in the naturalistic scenes simulated here, there was a range of positive SNRs over which comprehension was fully successful for our normally hearing listeners, yet listening was still perceived as being somewhat effortful. Importantly, this range of positive SNRs corresponds with the sort of listening conditions typically encountered in daily life (Smeds, Wolters, & Rung, 2015; Wu et al., 2017). Recent research in the field of listening effort emphasizes the importance of including test conditions corresponding to ecologically relevant signal-to-noise ratios, where effort may differ between listener groups despite intelligibility being near-perfect (Ohlenforst et al., 2017).

### Listening Is More Effortful in the Presence of Competing Speech

A further interesting finding that emerged from our analysis of listeners’ subjective ratings of effort and intelligibility is that, after controlling for better-ear SNR, listening was perceived as being significantly more effortful in scenes that contained competing speech compared with those that did not. The presence of competing speech did not have any corresponding effect on perceived intelligibility. We propose that greater perceived effort in the presence of competing speech could reflect an additional cognitive cost of overcoming informational masking (Kidd & Colburn, 2017). Parallels may be drawn to the findings of Koelewijn et al. (2012), who showed using pupillometry that, to maintain similar intelligibility levels, listeners must expend greater mental effort when listening to speech masked by another talker than by either stationary or fluctuating noise. In our study, greater perceived effort could conceivably reflect greater demand on attentional processes when competing speech is present in the auditory scene (Koelewijn, de Kluiver, Shinn-Cunningham, Zekveld, & Kramer, 2015).

### Limitations

Although we were able to successfully identify common intersubject brain activity in multiple frontotemporal regions, the absolute magnitude of the intersubject correlations that we observed was small (max ≈ 0.03). To some extent, this might reflect inherent limitations of the fNIRS technique, specifically its modest spatial resolution (Boas, Chen, Grebert, & Franceschini, 2004) and lack of an anatomical image. Despite best efforts to ensure consistent optode positioning across individuals, in the absence of an anatomical image of each individual’s brain, it is impossible to confirm that the fNIRS measurement channels were always similarly located with respect to underlying cortical anatomy. Thus, it is possible that ISC may have been reduced due to time courses from slightly different brain regions being compared across participants. Similarly, the modest spatial resolution of fNIRS may have weakened ISC due to blurring of activity across functionally distinct cortical regions (White et al., 2001). Although Pajula and Tohka (2014) have shown that ISC analyses are relatively forgiving of spatial smoothing as applied to fMRI data, fNIRS measurements inherently involve a much greater degree of spatial smoothing, which could obscure significant ISC occurring in localized subregions of the investigated cortical volume. This could be particularly problematic in the frontal cortex, where language-selective and domain-general subregions have been shown to lie in close proximity (Fedorenko, Duncan, & Kanwisher, 2012). Limited spatial resolution and potential intersubject differences in anatomical registration might similarly have hampered our attempts to identify significant relationships between brain activity and self-reported listening effort. In principle, these issues might be partly resolved in future studies by employing high-density diffuse optical tomography, an extension of fNIRS which uses spatially overlapping measurement channels at multiple source–detector distances to achieve higher spatial resolution (Chitnis et al., 2016).

Another factor that may have acted to increase variance in our brain-imaging data was our decision to include all eligible participants irrespective of handedness. It is common practice in cognitive neuroscience to exclude left-handed participants in order to reduce variance in the data, despite left-handedness occurring within a substantial portion (roughly 10%) of the population (Willems, der Haegen, Fisher, & Francks, 2014). Rerunning our analyses to include only right-handed participants (*N* = 22 of 30) did not reveal any fundamental changes compared with the results shown for the full sample, although in a few cases, individual-channel results that had been significant in the full sample became nonsignificant in the reduced analysis. This suggests that patterns of brain activity in the non-right-handed participants (four left handers and four having no strong preference for either hand) were sufficiently like those of the right handers to boost statistical power when included in the sample. This is perhaps not surprising given that, among left-handers, only a minority (roughly 25%) display atypical hemispheric dominance for language (Pujol, Deus, Losilla, & Capdevila, 1999; Szaflarski et al., 2002). The number of left-handed participants (*N* = 4) was unfortunately too small to analyze data for this subgroup in isolation.

A limitation of our experimental design is that different narratives were used for brain imaging and for subsequently obtaining participants’ subjective ratings of listening effort and intelligibility. This decision was necessitated by the requirement that all participants should be exposed to the same stimuli during brain imaging, a prerequisite for undertaking ISC analysis. We made efforts to match the stimulus used to obtain subjective ratings for a particular scene as closely as possible to that presented during brain imaging (same talker identity, duration of speech extract matched, and A-weighted SNR equalized). Nevertheless, differences in narrative content may have meant that, in some cases, the subjective listening experience in the subjective-ratings task was not fully representative of the experience when listening to the corresponding scene in the brain-imaging task. Any such differences would have manifested as a source of uncontrolled variance in our dataset.

A further limitation is that we did not have available any complementary measure of task engagement collected concurrently with fNIRS imaging (excepting the content-recognition questions that were presented after each narrative; for reasons already discussed, these questions did not provide an adequate indicator of sustained task engagement). Although participants only infrequently reported having felt compelled to give up listening in individual scenes, during the brain-imaging task, they were required to maintain attention for several minutes at a time. We cannot rule out that participants may have disengaged from listening attentively to the target speech at some points during the narratives. Indeed, anecdotally, several participants reported having struggled to maintain their attention for the full duration of the imaging session. Idiosyncratic lapses in attention or task engagement would be expected to reduce ISC, especially in higher order regions potentially involved in effortful speech comprehension. Similarly, any such lapses might also have hindered our attempts to correlate brain responses with perceived effort. In future research, it would be beneficial to combine fNIRS imaging with the simultaneous acquisition of other markers of physiological arousal, such as pupil dilation (McGarrigle, Dawes, Stewart, Kuchinsky, & Munro, 2017). Of course, pupil dilation is itself well established as a putative measure of listening effort (Zekveld & Kramer, 2014). Combined fNIRS or pupillometry studies could prove a powerful approach to shed light on the neural correlates of pupil dilation during effortful listening, especially under naturalistic conditions as considered here.

## Conclusion

When listening to continuous narratives in naturalistic scenes, participants perceived the target speech to become fully intelligible at a lower SNR than was needed for listening to become subjectively effortless. After controlling for better-ear SNR, self-reported effort was greater in the presence of competing speech, potentially reflecting an additional cognitive cost of overcoming informational masking. Using the optical brain-imaging technique fNIRS, we found evidence of common brain activity across individuals in multiple frontotemporal regions during listening to speech in naturalistic scenes. Also, activity in bilateral superior temporal cortex (i.e., the auditory cortices), as well as a region of right dorsolateral prefrontal cortex, significantly tracked the acoustic envelope of the target speech. However, we were unable to identify significant relationships between brain activity and self-reported listening effort or acoustic SNR. Further research is needed to fully understand the brain activity that underpins effortful listening in complex, everyday environments.

## Supplemental Material

Supplemental material for Listening in Naturalistic Scenes: What Can Functional Near-Infrared Spectroscopy and Intersubject Correlation Analysis Tell Us About the Underlying Brain Activity? Click here for additional data file. Supplemental material for Listening in Naturalistic Scenes: What Can Functional Near-Infrared Spectroscopy and Intersubject Correlation Analysis Tell Us About the Underlying Brain Activity? by Stephen C. Rowland, Douglas E. H. Hartley and Ian M. Wiggins: the MTN-017 Protocol Team in Trends in Hearing
