# Supplementary material for: Listening in Naturalistic Scenes: What Can Functional Near-Infrared Spectroscopy and Intersubject Correlation Analysis Tell Us About the Underlying Brain Activity?
Source: Trends Hear. 2018 Oct 22;22:2331216518804116. doi: 10.1177/2331216518804116 (PMC6198387; doi:10.1177/2331216518804116)
Supplement: Supplemental material for Listening in Naturalistic Scenes: What Can Functional Near-Infrared Spectroscopy and Intersubject Correlation Analysis Tell Us About the Underlying Brain Activity? [file Supplemental_material.pdf]

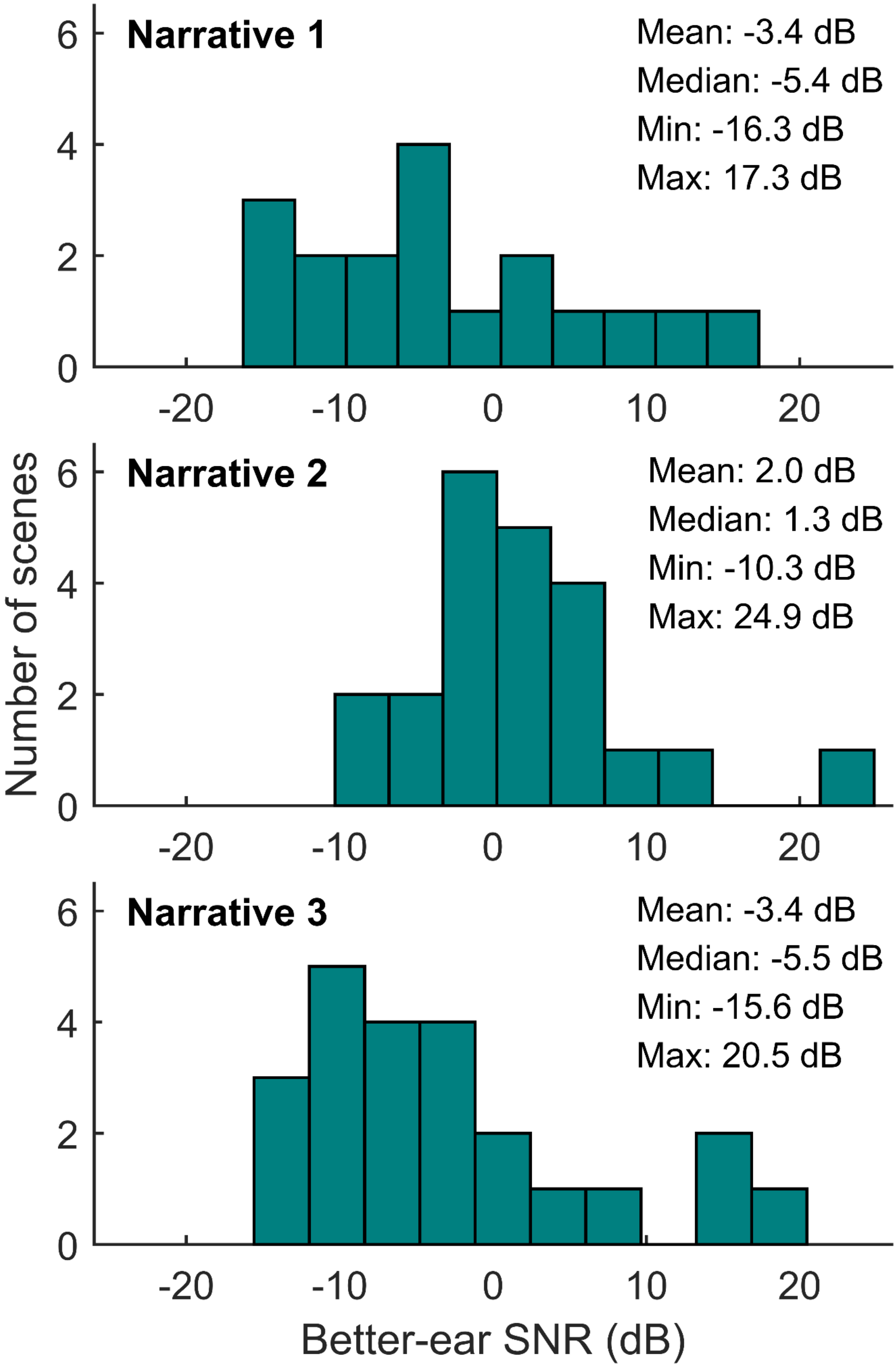

Supplementary Figure S1

Distributions of scene-by-scene better-ear SNR for the three narratives presented during brain imaging.
